# Sentinel lymph node B cells can predict disease-free survival in breast cancer patients

**DOI:** 10.1038/s41523-018-0081-7

**Published:** 2018-08-23

**Authors:** Kim R. M. Blenman, Ting-Fang He, Paul H. Frankel, Nora H. Ruel, Erich J. Schwartz, David N. Krag, Lee K. Tan, John H. Yim, Joanne E. Mortimer, Yuan Yuan, Peter P. Lee

**Affiliations:** 1 Department of Immuno-Oncology, City of Hope and Beckman Research Institute, Duarte, CA USA; 2 Department of Biostatistics, City of Hope and Beckman Research Institute, Duarte, CA USA; 3 Department of Pathology, Stanford University, Stanford, CA USA; 4 Department of Surgery, University of Vermont College of Medicine, Burlington, VT USA; 5 Department of Pathology, Memorial Sloan Kettering Cancer Center, New York, NY USA; 6 Department of Surgery, City of Hope and Beckman Research Institute, Duarte, CA USA; 7 Department of Women’s Health, City of Hope and Beckman Research Institute, Duarte, CA USA; 8 Present Address: Department of Dermatology, Yale University, New Haven, CT USA; 9 Present Address: Department of Pathology, Beaumont Health, Farmington Hills, MI USA

## Abstract

Tumor invasion into draining lymph nodes, especially sentinel lymph nodes (SLNs), is a key determinant of prognosis and treatment in breast cancer as part of the TNM staging system. Using multicolor histology and quantitative image analysis, we quantified immune cells within SLNs from a discovery cohort of 76 breast cancer patients. We found statistically more in situ CD3^+^ T cells in tumor negative vs. tumor positive nodes (mean of 8878 vs. 6704, respectively, *p* = 0.006), but no statistical difference in CD20^+^ B cells or CD1a^+^ dendritic cells. In univariate analysis, a reduced hazard was seen with a unit increase in log CD3 with HR 0.49 (95% CI 0.30–0.80) and log CD20 with HR 0.37 (95% CI 0.22–0.62). In multivariate analysis, log CD20 remained significant with HR 0.42 (95% CI 0.25–0.69). When restricted to SLN tumor negative patients, increased log CD20 was still associated with improved DFS (HR = 0.26, 95% CI 0.08–0.90). The CD20 results were validated in a separate cohort of 21 patients (*n* = 11 good outcome, *n* = 10 poor outcome) with SLN negative triple-negative breast cancer (TNBC) (“good” mean of 7011 vs. “poor” mean of 4656, *p* = 0.002). Our study demonstrates that analysis of immune cells within SLNs, regardless of tumor invasion status, may provide additional prognostic information, and highlights B cells within SLNs as important in preventing future recurrence.

## Immunology: Lymph nodes full of B cells linked to better outcomes

B cells within the tumor-draining lymph nodes may have an important biological role in preventing relapse of breast cancer. A team led by Peter Lee from City of Hope in Duarte, California, USA, quantified the levels of three populations of immune cells—T cells, B cells and dendritic cells—within sentinel lymph nodes biopsied from a cohort of 76 patients. They found that larger numbers of T cells and B cells were both linked to longer progression-free survival in the women. However, after statistically accounting for correlations between the two immune cell types, the researchers concluded that B cells had the dominant beneficial effect on survival times. They validated the finding that high B-cell counts are a prognostic indicator of better outcomes in a separate cohort of 21 women with triple-negative breast cancer.

## Introduction

Lymph node metastasis is a frequent early event in many cancers, forming one of three major factors in the TNM staging system. In breast cancer, lymph node invasion is a key determinant of risk and treatment. To reduce the morbidity associated with axillary lymph node dissection, sentinel lymph node (SLN) biopsy has replaced complete axillary lymph node dissection for many patients.^1–6^ It is also important to consider that lymph nodes are immune organs. As such, immune changes in lymph nodes may reflect disease progression and provide additional prognostic information. Our previous studies have shown that T cells and dendritic cells in axillary tumor-draining lymph nodes (TDLNs) may be altered in some breast cancer patients and can predict clinical outcome.^7–9^ B cells are another major immune cell population, but their role in cancer is less well studied. B cell infiltration into primary breast tumors and distant metastases is rare.^10^ When infiltration occurs, B cells in primary breast tumors have been shown to be clonally and functionally related to those in TDLNs.^11^ B cells isolated from TDLNs, specifically SLNs, can recognize cancer-associated antigens and are capable of producing antibodies against those antigens.^12,13^ In this study, we assessed the association of T cells, B cells, and dendritic cells within SLN with or without tumor invasion with disease-free survival (DFS) in breast cancer patients.

## Results

### SLN immune cells and tumor invasion

**Fig. 1:**
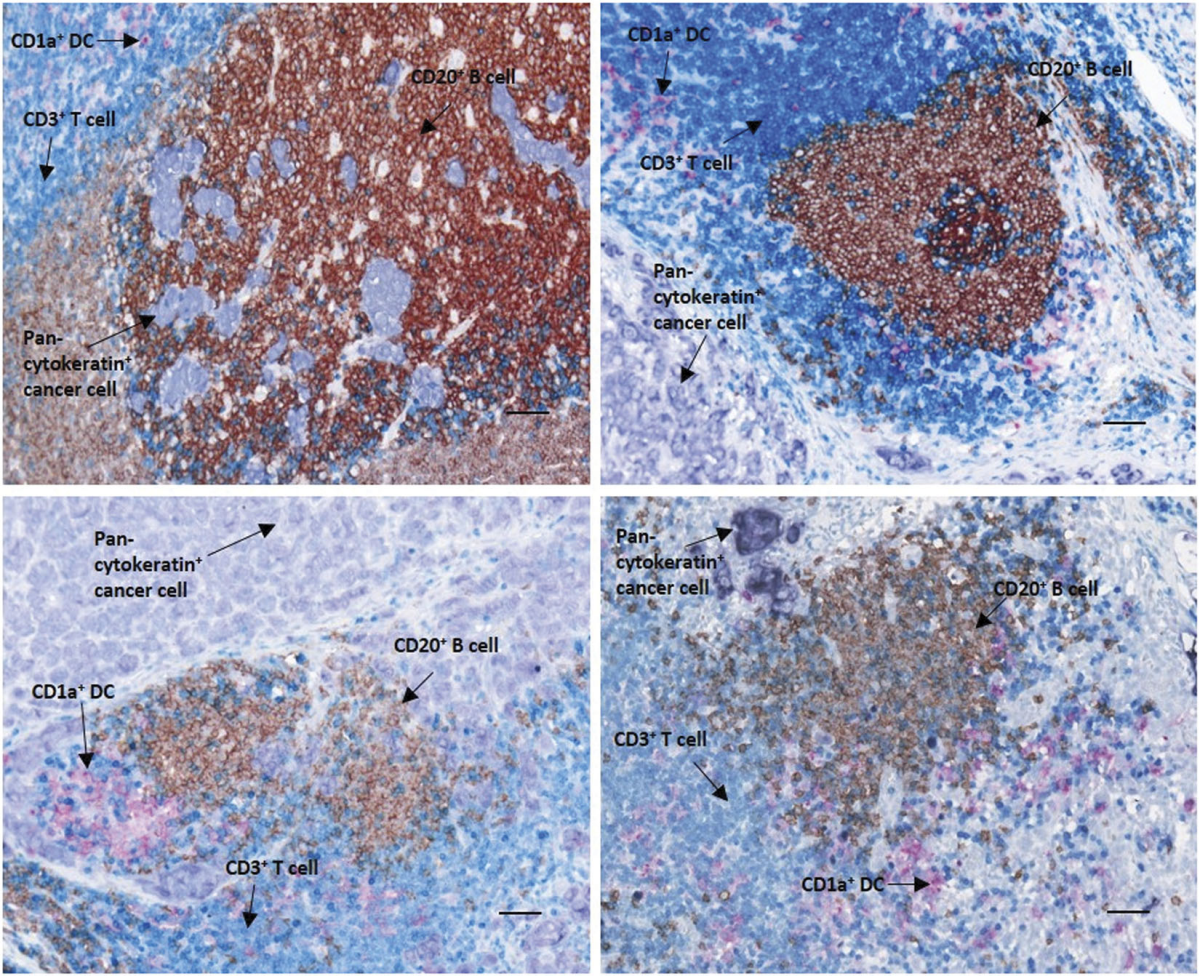
Five-color multiplexed chromogenic immunohistochemistry. FFPE samples were cut, stained with target antigens, and imaged at 20x magnification. Each panel represents a SLN from a single unique patient (*n* = 4) that displays all four targets of interest: CD3 T cells (blue), CD20 B cells (brown), CD1a dendritic cells (magenta), and pan-cytokeratin cancer cells (purple). Slides were scanned and quantitated using the Vectra™ Multispectral Quantitative Imaging System. Scale bar = 50 µm

**Fig. 2:**
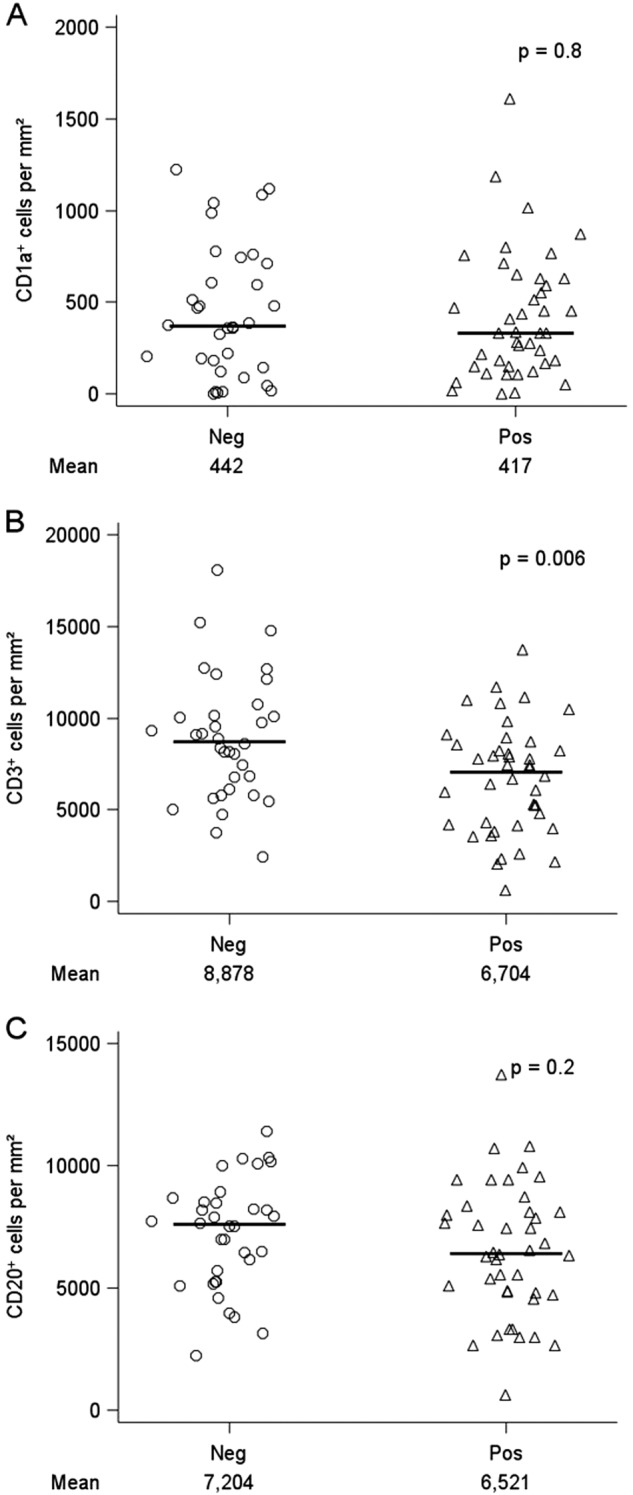
Immune cells in tumor invaded (pos) and tumor free (neg) SLNs. **a** CD1a^+^ dendritic cells, **b** CD3^+^ T cells, and **c** CD20^+^ B cells. *p*-values were from two-sided two-group *t*-tests on log-transformed values

We performed multiplexed IHC on formalin-fixed paraffin-embedded (FFPE) SLNs from 76 breast cancer patients (Table 1). Sample images of representative unique patients that display all four targets of interest: CD3 T cells, CD20 B cells, CD1a dendritic cells, and pan-cytokeratin cancer cells are shown in Fig. 1. We compared the numbers of CD3^+^ T cells, CD20^+^ B cells, and CD1a^+^ dendritic cells per mm^2^ area in tumor-invaded nodes (positive) to tumor-free lymph nodes (negative) (Fig. 2). In our cohort, we found a small but statistically significant reduction in the number of in situ CD3^+^ T cells (mean for tumor negative nodes of 8878 vs. tumor positive nodes of 6704, *p* = 0.006), but no statistically significant difference in CD20^+^ B cells or CD1a^+^ dendritic cells between tumor positive and negative SLNs (Fig. 2a–c).

**Table 1:** Patient characteristics

	DFS data set (*n* = 76)	TNBC data set poor outcome (*n* = 10)	TNBC data set good outcome (*n* = 11)
Age at diagnosis, median (range)	52 (29–77)	50.5 (35–65)	57 (43–72)
*ER/PR, n (%)*			
Neg	11 (17.1%)	10 (100%)	11 (100%)
Pos	56 (71.1%)	0 (0%)	0 (0%)
Unk	9 (11.8%)	0 (0%)	0 (0%)
*Her-2, n (%)*			
Neg	34 (44.7%)	10 (100%)	11 (100%)
Pos	11 (14.5%)	0 (0%)	0 (0%)
Unk	31 (40.8%)	0 (0%)	0 (0%)
Tumor size (cm), median (range)	1.9 (0.01–6.0)	1.5 (0.1–3.2)	3.2 (1.3–4.7)
*Tumor grade, n (%)*			
I	12 (15.8%)	0 (0%)	0 (0%)
II	33 (43.4%)	0 (0%)	2 (18%)
III	27 (35.5%)	10 (100%)	9 (82%)
Unk	4 (5.3%)	0 (0%)	0 (0%)
*Cancer stage, n (%)*			
I	20 (26.3%)	6 (60%)	1 (9%)
II	44 (57.9%)	3 (30%)	10 (91%)
III	10 (13.2%)	1 (10%)	0 (0%)
Other/Unk	2 (2.6%)	0 (0%)	0 (0%)
*Lymph node tumor status, n (%)*			
No tumor invasion	34 (44.7%)	10 (100%)	11 (100%)
Tumor invasion	42 (55.3%)	0 (0%)	0 (0%)

### Prediction of disease-free survival

In Table 2, we summarize the univariate and multivariate Cox regression results evaluating DFS based on SLN immune cells, tumor invasion, stage (stage 3 vs. all others), grade (grade 3 vs. all others), ER/PR+ vs. ER/PR−, age at diagnosis, and tumor size. In univariate analysis, SLN tumor invasion was associated with an increased HR 2.39 (95% CI 1.26–4.53), as was log CK with a HR of 1.14 (95% CI 1.02–1.28) and primary tumor size HR 1.36 (95% CI 1.10–1.69).

**Table 2:** Univariate and multivariate cox regression

Analysis of maximum likelihood estimates			Stepwise selection		Stratified by tumor status		Tumor negative only (*n* = 34)	
*n* = 76	Univariate		Multivariate		Multivariate		Univariate	
Parameter	Log-rank-P	HR (95% CI)	Wald-P	HR (95% CI)	Wald-P	HR (95% CI)	Log-Rank-P	HR (95% CI)
Tumor positive	<0.01	2.39 (1.26,4.53)	<0.03	2.10 (1.09, 4.04)	–	–	–	–
Stage 3	0.16	1.72 (0.80, 3.70)					0.45	2.14 (0.28–16.5)
Grade 3	0.18	1.51 (0.82, 2.79)					0.22	1.87 (0.66–5.29)
ER/PR+	0.60	0.81 (0.36, 1.83)					0.10	0.38 (0.11–1.26)
Age at dx (continuous)	0.19	0.98 (0.96,1.01)					<0.01	0.94 (0.90–0.98)
Tumor Size	<0.01	1.36 (1.10, 1.69)					0.03	2.09 (1.04–4.22)
logCK	0.03	1.14 (1.02, 1.28)					0.75	0.95 (0.68–1.32)
logCD1a	0.32	1.10 (0.91, 1.34)					0.40	1.13 (0.85–1.50)
logCD3	<0.01	0.49 (0.30, 0.80)					0.77	0.84 (0.27–2.62)
logCD20	<0.01	0.37 (0.22, 0.62)	<0.001	0.42 (0.25–0.69)	<0.001	0.39 (0.23–0.67)	0.03	0.26 (0.08–0.90)

**Fig. 3:**
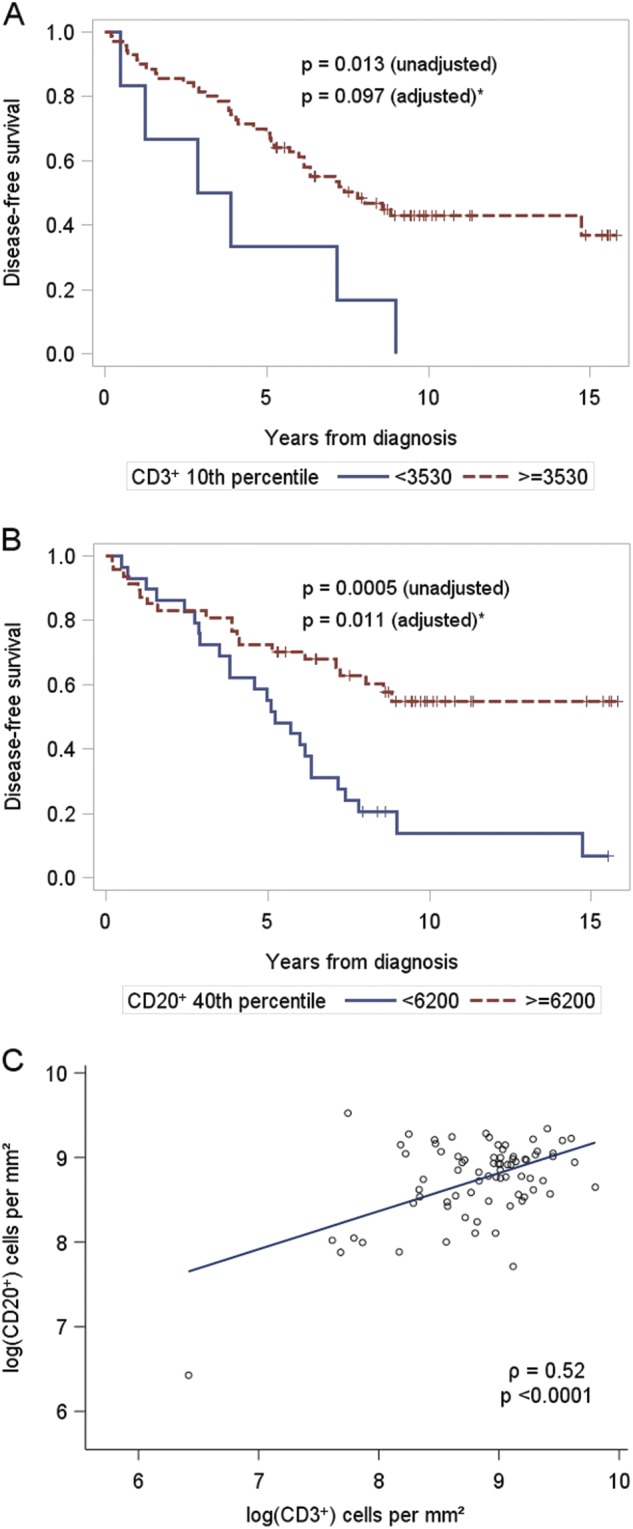
Disease-free survival based on immune cells in SLNs. Kaplan–Meier curves of the probability of DFS based on percentile cut-offs of **a** CD3^+^ T cell count and **b** CD20^+^ B cell count per mm^2^. Values below the percentile cut-off are depicted by a blue line. Values above the percentile cut-off are depicted by a red line. **c** Correlation between CD3^+^ T cell counts and CD20^+^ B cell counts in all SLNs. *Adjusted for the search for the best cut-point (see statistical analysis)

A reduced hazard was seen with a unit increase in log CD3 with HR 0.49 (95% CI 0.30–0.80) and log CD20 with HR 0.37 (95% CI 0.22–0.62). These results are based on CD3 and CD20 as continuous measurements. To display the impact graphically, the results for log CD3 and log CD20 are represented in Fig. 3a, b with Kaplan–Meier plots using cutoff-points for the number of CD3^+^ T cells (10th percentile), and CD20^+^ B cells (40th percentile) per mm^2^ area. While the actual statistical results are based on the continuous measurements, we also present the empirical *p*-value (unadjusted) associated with those best cut-offs (based on deciles), and a *p*-value adjusted for the multiple cut-point inflation of the Type I error.

For multivariate Cox regression, after backward stepwise regression, the two parameters that were retained in the model were SLN tumor invasion with HR 2.10 (95% CI 1.09–4.04) and log CD20 with HR 0.42 (95% CI 0.25–0.69). The *R*^2^ value for the 2-variable model was 0.2, and for tumor invasion alone 0.1. When stratifying on tumor invasion, the only parameter retained in the multivariable selection model was log CD20 (HR 0.39, 95% CI 0.23–0.67) further suggesting that B cells are highly associated with extended DFS.

To remove the potential effect of tumor invasion within SLNs, we further focused on the subset of SLN tumor negative patients (*n* = 34) within this cohort. We conducted a separate univariate analysis and found that increased log CD20 was still associated with improved DFS (HR = 0.26, 95% CI 0.08–0.90). Overall, these results suggest that patients with high numbers of either CD3^+^ T cells or CD20^+^ B cells in SLNs, regardless of tumor invasion, are less likely to relapse over time. T and B cells within SLNs are correlated (Fig. 3c, Pearson’s, *ρ* = 0.52, *p* < 0.0001), possibly explaining why both were not included in the final multivariate model of the full 76 patient cohort.

### Validation cohort and clinical outcome

TNBC is thought to be the subset of breast cancer patients most responsive to immunotherapy.^14^ In these patients, an understanding of the immune cell subsets may go beyond their prognostic value and could potentially lead to a large impact on future patient treatment decisions.^14^ In addition, there are still limited treatment options and TNBC patients are at the highest risk for early relapse.^15^ Approximately 34% of TNBC patients experience a distant recurrence with the average time of 2.6 years.^15^ However, the recurrence rate decreases sharply after 5 years.^15,16^ These factors motivated us to focus on this subset of patients for our validation cohort.

**Fig. 4:**
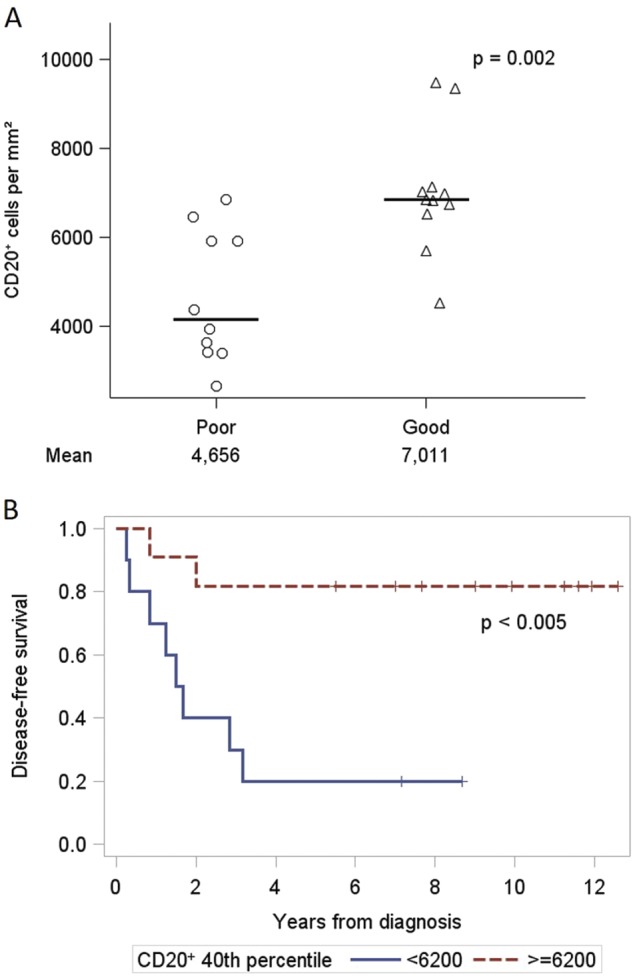
CD20^+^ B cells in tumor-free SLNs from validation set of 21 Triple-negative breast cancer patients. **a** Patients were considered either good outcome (DFS > 50 months) or poor outcome (DFS < 40 months). Statistics were based on a two-sided two-group *t*-test on log-transformed values. **b** Kaplan–Meier curves of the probability of DFS based on percentile cut-offs of CD20^+^ B cell count per mm^2^ established in the discovery cohort. Values below the percentile cut-off are depicted by a blue line. Values above the percentile cut-off are depicted by a red line

As a validation cohort, archived SLN FFPE samples from an additional 21 TNBC patients were analyzed. Patients were selected for having good outcome based on no progression with follow-up of >50 months, and poor outcome based on progression within 40 months. All of these patients had tumor negative SLNs. We hypothesized that if elevated CD20^+^ B cells are associated with better DFS in the original cohort consisting of different breast cancer subtypes and SLN statuses, this signal should be validated in a new cohort of patients of a single subtype and SLN status. This was the case, as the mean CD20^+^ B cells in SLNs for good outcome patients was 7011 per mm^2^, while the poor outcome patients mean was 4656 cells per mm^2^ (*p* = 0.002, Fig. 4a). We also applied the same threshold for CD20^+^ B cells (6200 per mm^2^ area) from the discovery cohort in Kaplan–Meier analysis of this validation cohort and found highly significant impact on clinical outcome with 5 year DFS of 20% for CD20 < 6200, and 81.8% 5 year DFS for CD20 ≥ 6200 (log-rank *p* < 0.005, Fig. 4b).

## Discussion

Mounting data demonstrate that the immune system is involved in successful control of cancer in some patients.^17^ Nature and degree of immune infiltration into tumors is now recognized as an independent prognostic factor,^18^ including for breast cancer.^19^ While tumor invasion into SLNs is a key determinant of prognosis, it is important to keep in mind that lymph nodes are immune organs. Alterations in immune profiles in TDLNs are becoming recognized as potential additive information. Through analysis of SLNs, our results suggest that the immune suppressive microenvironment extends beyond the tumor to draining lymph nodes. Important immune changes involve not only T cells, but also B cells and dendritic cells.^7–9^

Several studies have suggested that B cells play a role in controlling cancer.^10–12^ In a study of 1470 primary invasive breast carcinomas, higher total number of infiltrating CD20^+^ B cells was associated with significantly longer disease-free intervals.^20^ This benefit was independent of tumor grade, tumor size, cancer cell invasion status of the lymph nodes, and CD8^+^ T cell counts.^20^ Extending beyond the tumor, our data demonstrate that higher B cell numbers in SLNs are also associated with longer DFS. In our discovery cohort, T cells in SLNs were also associated with longer DFS in univariate analysis; however, only B cells in SLNs were associated with improved DFS in multivariate analysis. This may be partly due to the correlation between T and B cells within SLNs, with B cells having the dominant effect on survival.

Tumor invasion into SLN alone is not sufficient to predict DFS. Some patients with positive lymph nodes do not relapse, while some patients with negative lymph nodes have relapses. Therefore, in this study we also sought to determine if a better DFS model could be created by combining SLN tumor invasion status with immune cell profile. Our results showed that by adding in CD20^+^ B cells, DFS model prediction improved by two-fold over cancer cell invasion status alone, although we still only capture a minority of the source of variation in outcome. The role of CD20^+^ B cells was also apparent in the univariate analysis in patients with tumor-free SLNs.

To further support the prognostic significance of CD20^+^ B cells in SLNs, we evaluated a validation cohort of TNBC patients with tumor negative SLNs: one group relapsed within 40 months (poor outcome) and a second group selected with good outcome (>50 months DFS). We hypothesized that good outcome patients would have higher CD20^+^ B cells in their SLNs, regardless of tumor invasion. This was indeed observed, with a substantially higher average CD20^+^ B cell count in the good outcome patients. This is also consistent with previous work showing that in a specific subset of patients, the highest primary tumor B cell/plasma cell scores correlated with the best distant-metastasis free survival.^21^

Decrease in B cells within SLNs in poor outcome patients suggests that B cells play an important biological role in preventing future relapse. Studies have shown that antigen-driven B cells migrate into the breast tumor microenvironment, proliferate, undergo somatic hypermutation and affinity maturation.^22–24^ However, it is unclear as to the extent of this B cell activity in patients that have recurrences, distant metastasis, or poor disease-free and overall survival. Therefore, follow-up studies to test the function of B cells from breast cancer patients are required for better characterization of B cells in these patients.

Follow-up studies will also address some of our limitations. First, patients in the discovery cohort were diverse in their disease characteristics and not uniformly treated or followed. The validation data set, while having more homogeneous patient characteristics, were also not uniformly treated or uniformly followed. Although, both sample sets were collected based solely on availability, we expect limited bias since the immune cells were not known at the time of selection. We also recognize that the validation cohort was small, preventing multivariate adjustment for patient characteristics and that the survival separation based on CD20 cells might be exaggerated due to the dichotomy in patient selection. Nevertheless, the fact that these results were noted in a limited diverse set of patients in our discovery cohort and confirmed in our smaller validation study in TNBC patients, suggests that the impact of CD20 is substantial and can be observed even in small studies with diverse patient characteristics and treatments.

Our previous papers also reported that immune cells in TDLNs could predict clinical outcome in breast cancer.^7,9^ In those papers, T cells and dendritic cells rather than B cells were found to be the most significant predictors for clinical outcomes. However, those papers focused on non-SLNs (NSLNs) in SLN+ patients, as compared to SLNs in this paper. In the current era of SLN biopsy, few patients go on to full axillary lymph node dissection, so NSLNs are rarely available for analysis. Thus, it is important to understand the role of immune cells within SLNs in relation to clinical outcome.

In conclusion, in a discovery cohort of breast cancer patients with mixed subtypes and SLN status, high numbers per mm^2^ of T cells and B cells predicted longer DFS, with B cells having a stronger influence on DFS in multivariate analysis. Reduced numbers per mm^2^ of SLN B cells were associated with poor outcome in a validation cohort of SLN tumor-negative TNBC patients.

## Methods

### Patients

The initial study cohort to evaluate the immune correlates on disease-free survival (DFS) consisted of 76 breast cancer patients treated at Stanford University, University of Vermont Fletcher Allen Breast Care Center, Memorial Sloan Kettering Cancer Center, or City of Hope Comprehensive Cancer Center from 1994 to 2014. Patients were selected based on availability of archival lymph node tissues for research collected as part of the SLN biopsy. Patients ranged from 29 to 77 years of age (Table 1), with a median age of 52 years and excluded DCIS and Stage IV patients. For this cohort, one lymph node per patient was arbitrarily selected by staff in the respective Surgery or Pathology Departments for this study. The lymph node assayed for immune cell characteristics were tumor negative in 34/76 (45%) patients, and tumor positive in 42/76 (55%). Patient follow-up was based on standard of care. Median follow-up of event-free patients was 9.6 years, range 5.3–15.8. Initial diagnoses were made by core biopsy or by needle aspiration. Pathologic evaluation of the tissue was used to confirm final diagnosis. All samples were collected before therapeutic treatment. After surgical resection of the primary tumor, all patients received standard-of-care therapy as determined by their medical and radiation oncologists.

A separate validation cohort of a homogeneous group of triple-negative breast cancer (TNBC) patients seen at City of Hope consisted of 10 patients who relapsed within 40 months (poor outcome), and 11 patients who were relapse-free beyond 60 months (good outcome). These patients were also selected to have no tumor invasion in lymph nodes examined, and all lymph nodes examined were SLNs. Patients were diagnosed between 2002 and 2013 for the poor outcome patients with a median age at diagnosis of 50.5 years, range 35–65, and between 2002 and 2007 for the good outcome patients with a median age of 57 years, range 43–72. All TNBC patients were treated per standard of care at the City of Hope. SLNs were selected based on their designation as sentinel on the operative report. With all patients, cancer cell invasion status was determined by hematoxylin and eosin staining or IHC.

For both cohorts, the duration of DFS was the time between initial diagnosis and first recurrence. The biospecimens acquired from Stanford University (IRB #4947), University of Vermont (IRB #00000485), and City of Hope Comprehensive Cancer Center (IRB#12195 and #14346) were approved by the IRB boards from each of the respective institutions. The biospecimens obtained from Memorial Sloan Kettering Cancer Center were acquired through the Human Biospecimen Use Committee under the Memorial Sloan Kettering Cancer Center General Tissue Consent. The confidentiality of patients’ identifying information was protected at all times. All approved IRBs allowed a waiver of consent. Data gathered during this study did not influence the treatment or well being of the patients.

### Immunohistochemistry

Archived or fresh formalin-fixed paraffin-embedded biopsies of SLNs from breast cancer patients were sectioned and affixed to microscope slides. They were deparaffinized with xylene and rehydrated with decreasing concentrations of ethanol in water. Antigen retrieval was performed in a Digital Decloaking Chamber in DIVA Decloaker solution (Biocare Medical, Concord, California, USA). Tissue was stained with the following purified primary antibodies: mouse anti-human Cytokeratin (clone AE1/AE3; Biocare Medical); mouse anti-human CD20 (clone L26; Biocare Medical); rabbit anti-human CD3 (clone SP7; Biocare Medical), and mouse anti-human CD1a (clone CD1a007; Biocare Medical). Slides were subsequently stained with IgG secondary antibodies conjugated to alkaline phosphatase or horseradish peroxidase polymers. The antibody complexes were developed with diaminobenzidine (Biocare Medical), fast red (Biocare Medical), fast blue (Biocare Medical), NBT-BCIP (DAKO, Carpinteria, CA, USA), or VIP (Vector Laboratories, Burlingame, CA, USA). The cell nucleus was stained with hematoxylin (Biocare Medical).

### Multispectral imaging and quantitative analysis

Three to five-micron cuts of specimens were stained and scanned on the Vectra™ Multispectral Quantitative Imaging System (CRI/PerkinElmer, Hopkinton, Massachusetts, USA). Up to 98 sequential images were taken at 4x and up to 2450 sequential images were taken at 20×. Images were scanned at 10 nm interval wavelengths between 420 and 720 nm. This method generates quantitative spectral data for each pixel in an image with the help of Nuance™ analysis software (CRI/PerkinElmer, Hopkinton, Massachusetts, USA). Based on the spectral data, unique spectral profiles were created for each chromogen of interest. These profiles were combined into a spectral library specific for the image set. The spectral library allows for unmixing of each chromogen into independent channels, which allows for separation of colocalized or similarly colored chromogens. Once the images were unmixed, the number of specific immune cells per total nucleated cells were enumerated by InForm™ analysis software (CRI/PerkinElmer, Hopkinton, Massachusetts, USA) based on colocalization of markers of interest with the hematoxylin nuclear marker.

### Statistical analysis

Student *t*-test was used to compare in situ immune profiles between SLNs invaded by cancer cells and those that were cancer cell free. Log transformations were done for all immune marker values to reduce skewness.

Univariate and stepwise multivariate proportional hazards analyses were conducted to identify predictors of DFS, with each log-transformed immune variable analyzed as a continuous variable (no cut-points in Cox regression analysis). Univariate analysis was reported based on the log-rank statistics and hazard ratio and was conducted on all 76 DFS cohort patients, and separately on the subset of 34 patients who were tumor negative. For multivariate analysis, we conducted backward stepwise regression and reported on the hazard ratio (HR) and Wald statistic for the final parameters in the model. We also excluded tumor status from the stepwise regression and included it as a stratification factor. Cytokeratin breast cancer biomarker and all immune markers were considered for all predictive modeling. Immune cell count per mm^2^ area was used for the immune cell profiles.

Kaplan–Meier curves were based on specific cut-offs to illustrate differences in DFS. While the main results were based on the markers as continuous variables, we presented the log-rank statistics associated with the cut-offs chosen as best of the deciles examined. We also presented an adjusted statistic computed by adjusting for the multiple cut-point inflation of Type I error using resampling statistics. For this adjustment, 1000 resampled datasets were created in which the correlative variable was re-sampled to simulate no relationship between the correlative and outcome. The best cut-point procedure was re-run under this null model for each simulated dataset, and the *p*-value obtained was compared to the empirical *p*-value of the original data, where the percentage less than the empircal *p*-value is the adjusted *p*-value.

All statistical significance was considered as *p* < 0.05 (two-sided). Statistical analysis was conducted using SAS v9.4 and R Statistical software packages.

### Data availability

All data generated or analyzed in this study are included in this article.
